# Safety profile of a multimodal fail-safe model to minimize postoperative complications in oncologic colorectal resections—a cohort study

**DOI:** 10.1186/s13741-023-00291-6

**Published:** 2023-03-11

**Authors:** Shahram Khadem, Jonas Herzberg, Human Honarpisheh, Robert Maximilian Jenner, Salman Yousuf Guraya, Tim Strate

**Affiliations:** 1 Department of Surgery, Krankenhaus Reinbek St. Adolf-Stift, Hamburger Strasse 41, 21465 Reinbek, Germany; 2 Clinical Sciences Department, College of Medicine, University of Sharjah, P. O. Box 27272, Sharjah, United Arab Emirates

**Keywords:** Colorectal cancer, Perioperative complication, Anastomotic leakage, Colorectal resection, Multimodal approach, Laparoscopic surgery

## Abstract

**Background:**

Despite innovations in surgical techniques, major complications following colorectal surgery still lead to a significant morbidity and mortality. There is no standard protocol for perioperative management of patients with colorectal cancer. This study evaluates the effectiveness of a multimodal fail-safe model in minimizing severe surgical complications following colorectal resections.

**Methods:**

We compared major complications in patients with colorectal cancers who underwent surgical resections with anastomosis during 2013–2014 (control group) with patients treated during 2015–2019 (fail-safe group). The fail-safe group had preoperative bowel preparation and a perioperative single dose of antibiotics, on-table bowel irrigation and early sigmoidoscopic assessment of anastomosis in rectal resections. A standard surgical technique for tension-free anastomosis was adapted in the fail-safe approach. The chi-square test measured relationships between categorical variables, *t*-test estimated the probability of differences, and the multivariate regression analysis determined the linear correlation among independent and dependent variables.

**Results:**

A total of 924 patients underwent colorectal operations during the study period; however, 696 patients had surgical resections with primary anastomoses. There were 427 (61.4%) laparoscopic and 230 (33.0%) open operations, while 39 (5.6%) laparoscopic procedures were converted. Overall, the rate of major complications (Dindo-Clavien grade IIIb–V) significantly reduced from 22.6% for the control group to 9.8% for the fail-safe group (*p* < 0.0001). Major complications mainly occurred due to non-surgical reasons such as pneumonia, heart failure, or renal dysfunction. The rates of anastomotic leakage (AL) were 11.8% (22/186) and 3.7% (*n* = 19/510) for the control and fail-safe groups, respectively (*p* < 0.0001).

**Conclusion:**

We report an effective multimodal fail-safe protocol for colorectal cancer during the pre-, peri-, and postoperative period. The fail-safe model showed less postoperative complications even for low rectal anastomosis. This approach can be adapted as a structured protocol during the perioperative care of patients for colorectal surgery.

**Trial registration:**

This study was registered in the German Clinical Trial Register (Study ID: DRKS00023804).

## Introduction

The incidence of postoperative complications after colorectal surgery varies substantially, ranging from approximately 20% (Veldkamp et al. 2005) to 60% (Kristjansson et al. 2010). This vast variation in incidence of postoperative complications is due to the absence of a standard tool for grading the severity of complications and the use of different severity grading systems such as the Accordion grading system (Strasberg et al. 2009) and the Dindo-Clavien classification (Dindo et al. 2004). Additionally, some investigators include only the postoperative period, while others consider intraoperative complications as well (Kazaryan et al. 2013). Generally, complications following colorectal surgery can be caused by intraoperative and postoperative factors. Intraoperative complications such as hemorrhage, bowel injury, ureteral and bladder injuries result from intra-abdominal adhesions, anatomic difficulties, and essentially depend on the training and experience of the operating surgeon (Forgione and Guraya 2017; Guraya et al. 2015). Common postoperative complications include surgical site infection, anastomotic leakage (AL), paralytic ileus, and bleeding (Vather et al. 2015). Age, gender, nature, and stage of colorectal cancer are non-modifiable risk factors for postoperative complications (Guraya and Murshid 2004). Conversely, certain other perioperative modifiable risk factors that influence the outcome of colorectal surgery can be conveniently modified to mitigate the risk of surgical complications (Kirchhoff et al. 2010).

Apart from the scarcity of a unified tool to grade and report postoperative complications, an absence of a standard perioperative protocol for colorectal surgery amplifies the complexity in managing patients with colorectal cancer. Nevertheless, several popular protocols and pathways have been reported in the literature. The Early Recovery After Surgery (ERAS) pathway offers a reduction in perioperative stress and has been shown to be associated with improved 5-year cancer-specific survival after colorectal surgery (Gustafsson et al. 2016). In their case–control study, Arrick et al. have reported a significant reduction in both complication rates (31.5% vs. 14.6%; *p* ≤ 0.05) and mean length of hospital stay (10.1 vs. 6.9 days; *p* ≤ 0.05) using ERAS pathway (Arrick et al. 2019). However, the authors could not report patients‘ satisfaction rates or patients‘ reported outcomes in their data. Fast-track surgery (FTS) provides another set of evidence-based standards that can potentially reduce the physiological burden of surgery and can thus improve postoperative quality of life (Grant et al. 2017). FTS advocates perioperative measures for appropriate selection of surgical incision and anesthesia agents, reduces postoperative pain, and optimizes organ functions by mitigating physiological and psychological stresses associated with operations. In a meta-analysis of the clinical trials about the impact of ERAS/FTS on postoperative infections, Grant et al. have reported a significant reduction in postoperative lung infection [risk ratio (RR) = 0.38; 95% confidence interval (CI) = 0.23–0.61; *p* < 0.0001; *I* = 0%], urinary tract infection (RR = 0.42; 95% CI = 0.23–0.76; *p* = 0.004; *I* = 0%), and surgical site infection (RR = 0.75; 95% CI = 0.58–0.98; *p* = 0.04; *I* = 0%) as compared to controls (Grant et al. 2017).

Unfortunately, no single protocol for perioperative management of patients with colorectal cancer comprehensively elucidates pre-, peri-, and postoperative steps that can lead to early recovery and reduction of complications. We report the results of a multidisciplinary fail-safe model as an adoption of the known ERAS/FTS models, which aims to mitigate the rates of postoperative complications and to foster early recovery of the patients following operations for colorectal cancers.

## Methods

We enacted several modifications in a perioperative protocol for patients undergoing operations for colorectal cancer at hospital Reinbek St. Adolf-Stift, Germany. From January 2015 to December 2019, we prospectively collected data of patients with colorectal cancers who were consecutively treated with a multi-modal fail-safe approach of open and laparoscopic oncological resections at the study center. We excluded patients with benign colorectal lesions, those with surgical resections of colorectal cancer with terminal stoma, and patients who had surgery for associated malignancies such as ovarian or pancreatic cancers. In the first months of 2015, while establishing the multimodal “fail-safe“ approach, colorectal resections were performed by two experienced colorectal surgeons (> 50 colorectal resections per year). We adapted a system that ensured the presence of an experienced surgeon (> 30 resections with anastomosis per year) for every colorectal resection. However, a number of surgical colorectal procedures were performed by senior surgical residents under the guidance and supervision of an experienced colorectal surgeon. The surgical team comprised an experienced colorectal surgeon and an experienced colorectal resident or fellow, assisting more than 20 colorectal resections per year. According to the American Joint Committee on Cancer and the Union for the International Cancer Control AJCC/UICC) classifications, the patients with rectal cancer of the middle (> 6–12 cm from the anal verge) or lower third of the rectum (< 6 cm from the anal verge) received neo-adjuvant therapy if staged UICC IIA or higher (Edge and Compton 2010).

### Control group

As a control group, we retrospectively analyzed all patients with colorectal cancer who underwent oncological colorectal resections from January 2013 to December 2014 using the same exclusion criteria. Previous data were incomplete due to a change in the documentation system in the hospital. Although colorectal resections during the defined period were either performed or supervised by an experienced colorectal surgeon, no structured protocol was followed. Nevertheless, some components of the fail-safe protocol were sporadically adapted in a small group of patients within the control group (perioperative intravenous antibiotics, experienced surgeon). The patients were generally treated using the ERAS protocol without preoperative colonic irrigation. In the control group, we did not routinely perform perfusion tests before anastomosis as there was no standard approach for anastomoses, which ranged from end-to-end in right hemicolectomy to side-to-end in rectal resections. Similarly, in rectal resections, no routine ileostomy or on-table lavage was performed.

### Fail-safe group

Since January 2015, we adapted a standard protocol for the perioperative management of patients with colorectal cancers. This includes a mechanical bowel preparation without oral antibiotics prior to any kind of planned colorectal surgery and routine postoperative nutritional care with oral liquids including high-protein intake for three days after surgery. A step-wise description of surgical procedures in the fail-safe group is outlined hereunder.

### Surgical approach and technical steps in the fail-safe group

For the right-sided tumors, a complete mesocolic excision (CME) with a primary central vascular ligation (CVL) and a D3 lymphadenectomy was routinely performed (Guraya et al. 2005; Kirchhoff et al. 2010). Likewise, resection of the left-sided tumors was performed using the core principles of surgical oncology for CME and CVL (high-tie) (Guraya 2016). A detailed protocol for the multimodal fail-safe approach is outlined in Table 1.

**Table 1:** Protocol of multimodal fail-safe approach for colorectal resections in our study

**Preoperative settings**	
Bowel preparation (for right-, left-sided, and rectal resections)	O
Preoperative intravenous antibiotics	O
**Operative approach/technical aspects**	
Experienced colorectal surgeon	O
Complete mobilization of the hemicolon for tension-free anastomosis	O
Bleeding/perfusion test at the edge of the resection margin	O
Side-to-side anastomosis	
- Continuous seromuscular suture	O
- Additional seams on the edges to relieve tension on the anastomosis	O
End-to-end anastomosis	
- Mesentery is in line with the resection margin	O
- Do not free endings from fatty tissue	O
- Avoid sharp-angled edges	O
- Stretching of the anal sphincteric muscle for three minutes	O
- Spine of the stapling device next to the stapled line	O
- After joining ends, compression for at least one minute before release	O
- Anastomotic assessment using sigmoidoscope (air test + intraluminal inspection)	O
- Diverting stoma for low rectal anastomosis	O
- On-table lavage over efferent loop of ileostomy with 5L of saline	O
- Placement of a drainage tube near the anastomosis	O
**Postoperative settings**	
- A 3-day low-volume high-calorie nutrition (except patients with diverting stoma)	O
- Full meals from 4^th^ postoperative day onwards	O
- Endoscopic control of colorectal-/coloanal anastomosis on 4^th^ postoperative day	O
- In case of suspected anastomotic leakage, over-the-scope-clip application	O

**Fig. 1:**
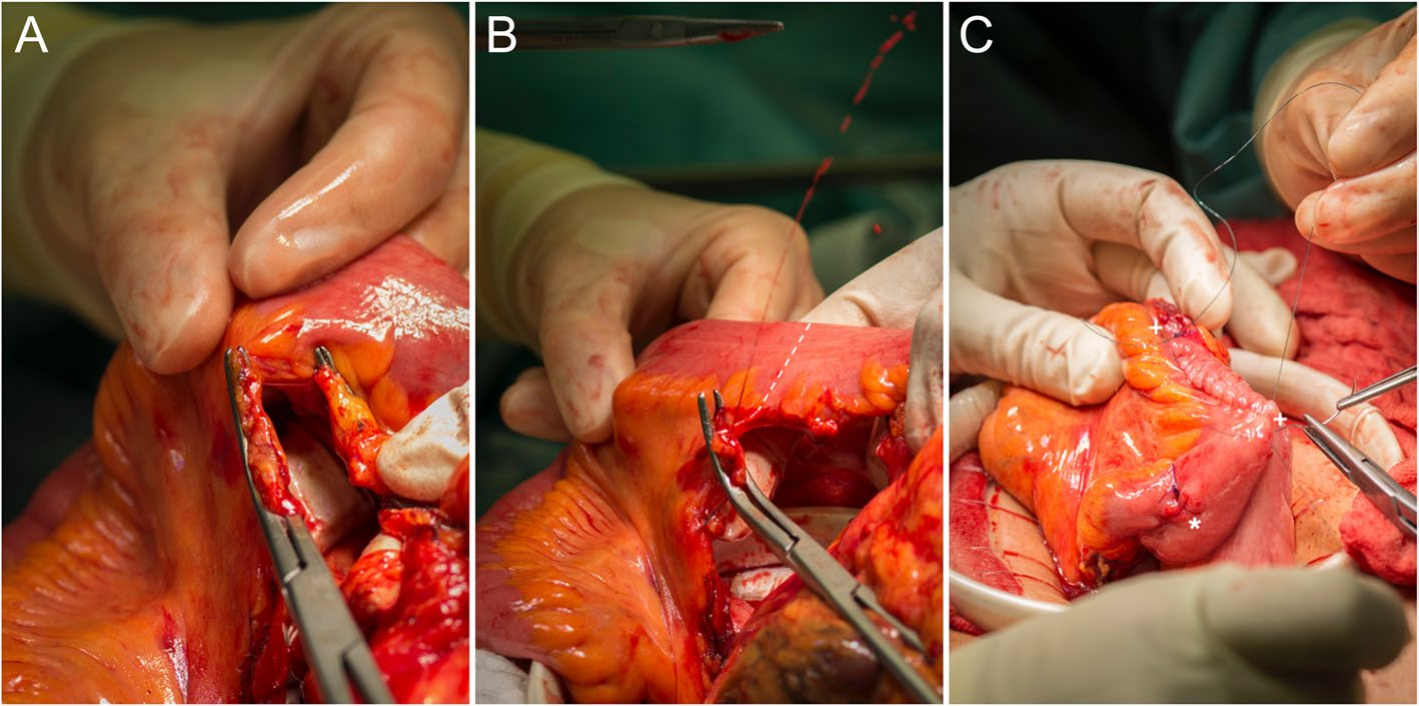
**A**, **B** Blood perfusion testing at the edge of resection. Line marking resection margin. **C** Iso-peristaltic side-to-side anastomosis with additional seams on both edges ( +) for mechanical strain relief. Stapled endings are also oversewn (*)

**Fig. 2:**
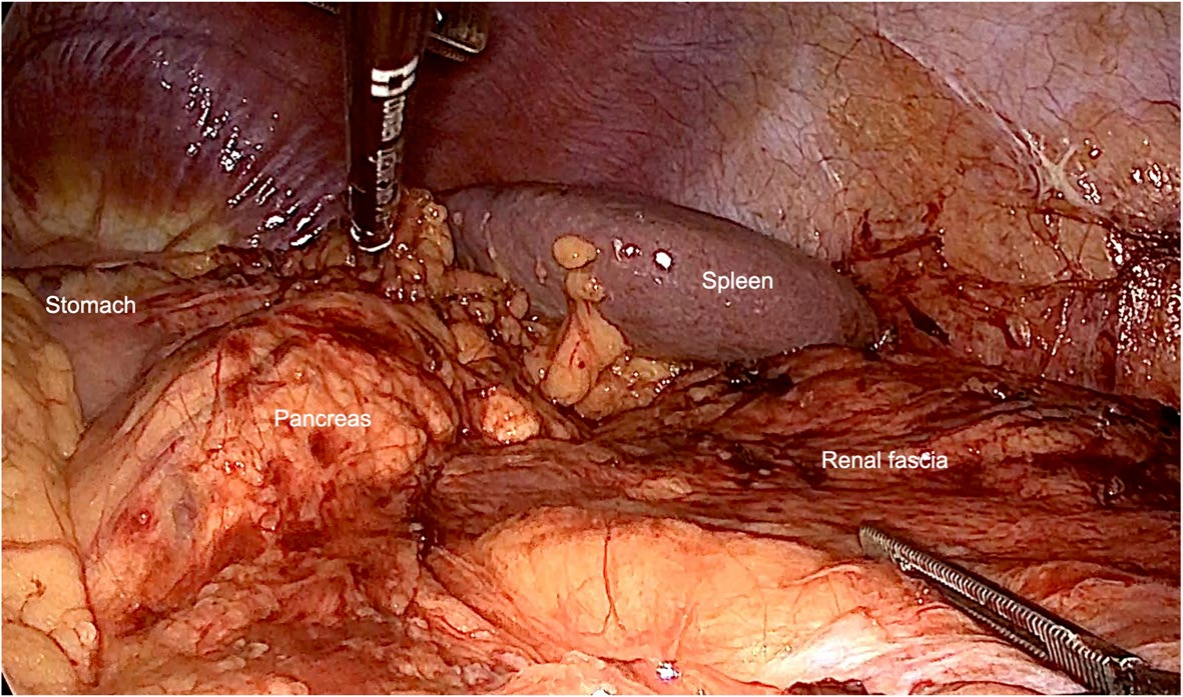
For the left-sided or rectal resections, complete mobilization of the colon up to the middle colic artery for tension-free anastomosis

**Fig. 3:**
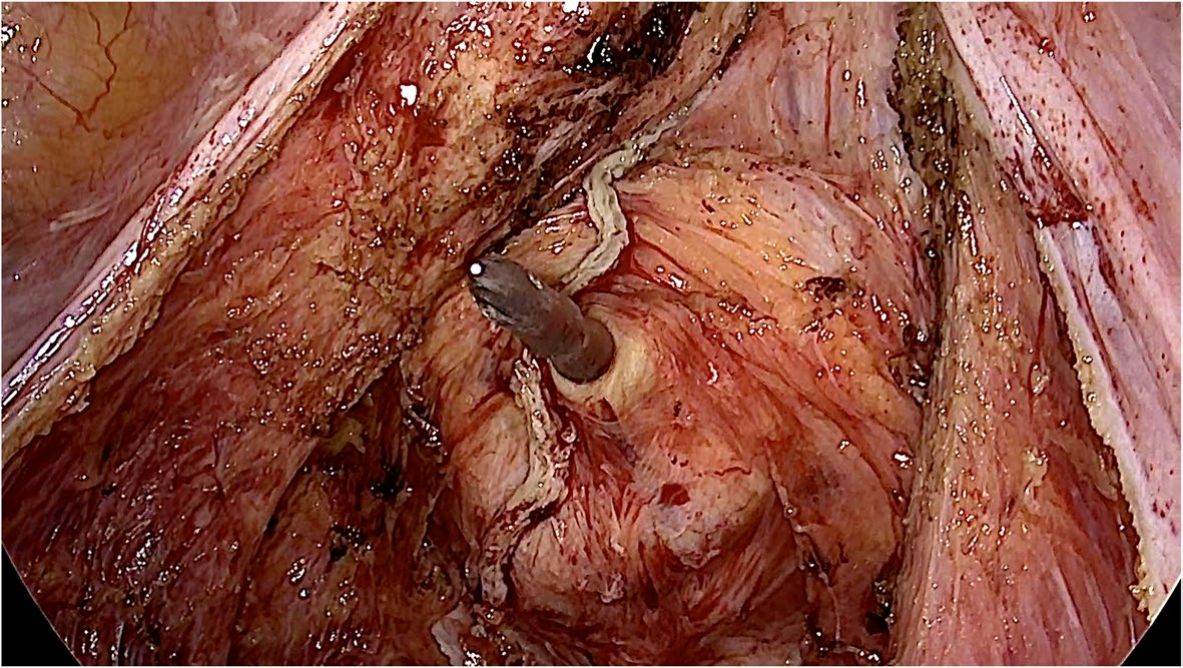
The rectal stump covered with fatty tissue. Spine of the stapler is piercing out of the rectal stump

For the ileocolonic anastomosis, a hand-sewn continuous technique was preferred. Furthermore, we performed additional seams on the edges of side-to-side anastomosis, potentially reducing tangential stress on anastomotic sutures caused by normal bowel movement (Fig. 1C). The bleeding test for confirmation of arterial perfusion was performed immediately at the edge of the resection margin (Fig. 1A, B). Avoiding sharp edges and leaving fatty tissue around edges preserves microperfusion at the high-risk anastomotic zone. Impaired microperfusion leads to tissue necrosis and anastomotic leakage. For the colorectal or coloanal anastomoses, a full mobilization of the splenic flexure up to the middle colic artery was performed (Fig. 2). For rectal cancer, after nerve preserving total mesorectal excision (TME), dissection was performed below the tumor at a level of 1–3 cm from the anal verge. When performing the anastomosis, the spine of the stapler was stuck out next to the staple line of the distal rectum (Fig. 3). Otherwise, overlapping of circular and straight stapling lines of the rectum over a long distance would increase the risk for vascular insufficiency. Additionally, this step prevents possible dislocation of metal staples in the anastomotic area, thus mitigating the risk of incomplete anastomotic closure. After joining proximal and distal resection margins, compression of the endings was essential for at least one minute before the release of stapling device, ensuring that the fatty tissue in between is flattened to a maximum for perfect seromuscular anastomosis. A partial mesorectal excision (PME) was performed for tumors of the sigmoid colon and upper rectum (12–16 cm from the anal verge). Furthermore, in some emergencies, a decision for a protective ileostomy was made due to an unprepared colon. Even after preoperative irrigation, the colon is usually not fully cleaned and leftovers are still in place. Besides preoperative bowel preparation, intraoperative colonic irrigation via the efferent loop of the ileostomy was performed with 5L of saline for all patients with the need for a protective ileostomy. All patients with low rectal anastomosis received protective ileostomy. In the case of a rectal resection, endoscopical control was performed on the fourth postoperative day to exclude the possibility of an occult anastomotic leakage.

A pelvic drain was placed around anastomosis that served to drain the pelvic abscess in case of AL. Furthermore, this drainage tube serves as a marker for anastomotic insufficiency, when performing intraoperative colonic irrigation with saline. Multivisceral resections were defined as any further resection of the intestine or other organs apart from the main tumor resection. This ranged from concurrent cholecystectomy, adrenalectomy, appendectomy, or resection of the urinary bladder dome up to major multivisceral resections including hepatic, pancreatic, and gastric resections.

### Patients’ characteristics and definitions

We collected data about the patients’ demographics, body mass index (BMI), the American Society of Anesthesiologists (ASA) classification of physical health, tumor localization, surgical approach and procedure, length of hospital stay, complications according to Dindo-Clavien’s classification (Dindo et al. 2004) and mortality. Postoperative morbidity was defined as a complication occurring within 90 days after surgery. A 90-day mortality rate is reported to depict the postoperative outcome in a proper way (Byrne et al. 2013). Patients were staged according to the AJCC/UICC classification for colorectal cancer (Edge and Compton 2010). AL was defined as a defect of the intestinal wall integrity at the ileocolic, colorectal or coloanal anastomotic site including suture and staple lines of neorectal reservoirs leading to a communication between the intra- and extraluminal compartments (Parthasarathy et al. 2017). We used early endoscopy to detect AL and performed CT scan after endoscopy, if needed. A pelvic abscess close to the anastomosis was also considered as AL. Although there is no universal grading, leakages were graded according to the system proposed by Rahbari et al. (Rahbari et al. 2010).

### Statistical analysis and ethical approval

Statistical analyses were performed using IBM SPSS Statistics Version 25 (IBM Co., Armonk, NY, USA). All variables were listed as means with standard deviation. Categorical variables were arranged as numbers with percentages. The chi-square test was used to determine relationships between categorical variables and *t*-test was used to measure the probability of difference between two sets of data. In the multivariate analysis, we used logistic regression analysis for predicting AL in standard and fail-safe groups by controlling variables such as old age, days in hospitals, survival up to 90 days, and surgical site infection (SSI). Logistic regression was applied as dependent variable as a binary variable, valued as 1 for AL and 0 for non-leakage. A *p*-value less than or equal to 0.01 and 0.05 was considered statistically significant.

This study was registered in the German Clinical Trial Register and conducted in accordance with the declaration of Helsinki. As this is a retrospective anonymized analysis, no additional written consent and ethical approval was needed.

The data is reported according to the STROCSS guideline (Agha et al. 2019).

## Results

During the study period, 924 patients underwent colorectal resections for UICC stage I–IV colorectal cancers and other malignancies from January 2013 to December 2019. Of these, 228 patients did not have anastomosis due to certain reasons; infiltration of the sphincter muscle, palliative resection, or immobilization. A detailed analysis of 696 patients who fulfilled our study criteria is shown in Table 2.

**Table 2:** Characteristics of the patients in our study with colorectal cancers (*n* = 696)

	**Total**	**Control group** ***n* = 186**	**Fail-safe group** ***n* = 510**	***p*-value**
Age (mean), years		70.63	69.79	NS
Sex (%)				
Male	375 (53.9)	99 (53.2)	276 (54.1)	NS
Female	321 (46.1)	87 (46.8)	234 (45.9)	NS
ASA classification (%)				
ASA 1	57 (8.2)	13 (7)	44 (8.6)	NS
ASA 2	326 (46.8)	82 (44.1)	244 (47.8)	NS
ASA 3	295 (42.4)	89 (47.8)	206 (40.4)	NS
ASA 4	18 (2.6)	2 (1.1)	16 (3.1)	NS
BMI (mean), kg/m^2^		25.62	26.71	NS
Indication for surgery (%)				
Colon cancer	572 (82.2)	143 (76.9)	411 (80.6)	NS
Left side	186 (26.7)	56 (30.1)	130 (25.5)	NS
Right	330 (47.4)	77 (41.4)	253 (49.6)	NS
Other parts	56 (8.0)	12 (6.5)	44 (8.6)	NS
Rectal cancer	124 (17.8)	41 (22.0)	83 (16.3)	NS
UICC/AJCC (%)				
0	9 (1.3)	1 (0.5)	8 (1.6)	
I	207 (29.7)	62 (33.3)	145 (28.4)	
II	217 (31.2)	45 (24.2)	172 (33.7)	
III	202 (29.0)	54 (29.1)	148 (29.0)	
IV	61 (8.8)	24 (12.9)	37 (7.3)	
Surgical approach (%)	< 0.0001			
Open	230 (33)	96 (51.6)	134 (26.3)	
Laparoscopic	427 (61.4)	74 (39.8)	353 (69.2)	
Conversion	39 (5.6)	16 (8.6)	23 (4.5)	
Protective ileostomy (%)	128 (18.4)	39 (21)	89 (17.5)	NS
Multivisceral resection (%)	124 (17.8)	50 (26.9)	74 (14.5)	NS

*ASA* American Society of Anesthesiologists, *BMI* Body mass index, *UICC/AJCC* The Union for International Cancer Control/The American Joint Committee on Cancer, *NS* Not significant

There were 375 (53.9%) men and 321 (46.1%) women with a mean age of 70.21 years. A maximum number of 326 (46.8%) patients belonged to ASA 2, while the average BMI of patients in our series was 26.1 kg/m^2^. The data included 427 (61.4%) laparoscopic and 230 (33.0%) open procedures, while 39 (5.6%) laparoscopic procedures were converted to open surgeries. There were 186 patients in the control group and 510 patients in the fail-safe group; 572 (82.2%) colon cancers (186 left-sided and 330 right-sided) and 124 (17.8%) rectal cancers. Apart from different surgical approaches, both groups showed no significant differences in their demographics.

In our study, the overall rate of AL in the fail-safe group (Table 3), including emergency surgeries and multivisceral resections, was 3.7% (*n* = 19). There was a significantly less incidence of AL in the left-sided than the right-sided colonic resections (*n* = 2/130 vs 4/56,* p* = 0.047) and lower rectal resections (*n* = 1/83 vs 15/41, *p* < 0.001). In the right-sided colonic resections, no significant difference was found. We performed a subgroup analysis for colorectal and rectal resections showing a significant lower AL rate in rectal surgery (*p* < 0.001) and an insignificant lower AL rate for colonic resections (4.4% vs. 4.9% in the control group, *p* = 0.814). The only AL following a rectal resection was diagnosed and treated by endoscopy.

**Table 3:** Comparison of surgical outcomes between fail-safe and control groups within the first 90 days after surgery (*n* = 696)

	**Control group** ***n* = 186**	**Fail-safe group** ***n* = 510**	***p*-value**
Major complication DC ≥ 3b (%)	42 (22.6%)	50 (9.8%)	< 0.0001
Re-operation (%)	35 (18.8%)	31 (6.1%)	**< **0.0001
Pneumonia (%)	12 (6.5%)	13 (2.5%)	0.014
Wound infection (%)	34 (18.3%)	27 (5.3%)	** <** 0.0001
90-day mortality (%)	5 (2.7%)	12 (2.4%)	0.800
Anastomotic leakage (%)	22 (11.8%)	19 (3.7%)	** <** 0.0001
Left colon resection	4	2	0.047
Right colon resection	3	12	0.755
Resection of other parts of the colon	0	4	0.278
Low rectal resection	15	1	** <** 0.001

*DC* Dindo-Clavien classification

In the fail-safe protocol, using the Dindo-Clavien’s classification, we found postoperative complications in 150 patients (29.4%) with a major complication rate (Dindo-Clavien ≥ 3b) of 9.8%; 360 (70.6%) patients did not have any complication, grade I: 52 (10.2%); grade II: 38 (7.5%); grade IIIa: 10 (2%); grade IIIb: 32 (6.3%); grade IV: 6 (1.2%); and grade V: 12 (2.4%). Following the subgroup analysis for colorectal and rectal surgery, the major complication rate could be reduced significantly using the fail-safe approach (colorectal surgery: *p* = 0.012; rectal surgery: *p* < 0.001). The mortality rate in our study was 2.4% (*n* = 12) in the fail-safe group and 2.7% (*n* = 5) in the control group (*p* = 0.80).

Table 4 provides an overview of the causes of postoperative mortality. In our study, the average postoperative hospitalization time was 8.9 days.

**Table 4:** Causes for postoperative mortality in our study (*n* = 696)

	**Control group (*n* = 186)**	**Fail-safe-group (*n* = 510)**
Surgical complication	0	2
Internal complication		
Heart failure	0	1
Respiratory failure	0	3
Stroke	1	0
Sepsis	2	4
Unclear/other	2	2

The postoperative hospitalization time was significantly shorter in the fail-safe group (control group 18.9 days, *p* < 0.0001). The results of logistic regression analysis elicit significantly less leakage in a fail-safe group with *p* < 0.000 (Table 5). For the control variables, results showed that AL was significantly associated with longer hospital stay (*p* < 0.000), poor 90-day survival (*p* < 0.05), high surgical site infection (*p* < 0.000), and high re-operation rates (*p* < 0.000).

**Table 5:** Logistic regression analysis of the anastomotic leakage with fail-safe and standard approaches and control variables (*n* = 696)

Variable	Coeff	S.E	Wald	Sig
**Surgery type**				
Fail-safe	− 1.299***	0.330	15.468	0.000
Standard	− 0.244	0.444	0.302	0.583
**Control variables**				
Old age	− 0.029	0.015	3.766	0.052
Days in hospital	0.055***	0.010	32.335	0.000
90-day survival	− 1.149**	0.560	4.210	0.040
Surgical site infection	− 1.244***	.137	82.873	0.000
Re-operation	− 4.232***	.608	48.508	0.000
**Constant**	2.009***	.227	78.278	0.000

**p < 0.05

***p < 0.01

## Discussion

Our study showed a significantly low rate of major complications of 9.8% in colorectal resections following the fail-safe approach. Additionally, the rate of AL, including emergency surgeries and multivisceral resections, was as low as 3.7%, while AL was 1.2% for the planned low rectal resections (*n* = 1/83). There is often a debate about the accuracy of major surgical complications after colorectal resections as several studies exclude emergency operations, low rectal resections with anastomosis, and protective ileostomy or even include resections without anastomosis. A range of AL rates from 1.6% to 19.1% is reported in the literature (Hyman et al. 2009). The reported rates of major complications after colorectal resections with anastomosis range from 2.8% (Meylemans et al. 2018) to 12.3% (Park et al. 2016). In the forthcoming sections of the article, pursuing a multimodal fail-safe approach, this article provides a step-wise account of the protocol and justifications for each measure.

### Preoperative settings

Literature has shown that a preoperative inflammatory event leads to a higher risk of postoperative infectious complications (De Magistris et al. 2016). In our study, besides a preoperative mechanical bowel preparation, an intraoperative single shot-antibiotic, half an hour prior to incision was administered to all patients. Recent studies have even advocated a combination of preoperative oral antibiotics, mechanical bowel preparation, and intravenous antibiotics at induction (Scarborough et al. 2015). However, due to a low rate of postoperative infectious complications in our study, we did not find any benefit of adding an oral antibiotic prior to operation. Conversely, we performed an adequate preoperative bowel irrigation as the presence of stools causes intraluminal bacterial contamination, increased mechanical intraluminal pressure, and shear stress on the anastomosis.

### Surgical approach and technical aspects

Generally, the surgeon’s responsibility for postoperative complications is often denied, and the responsibility is placed on patient-related risk factors, notably to male gender (Sciuto et al. 2018), neoadjuvant (radio-)chemotherapy (Swellengrebel et al. 2011), lack of mechanical bowel preparation and decontamination (Murray and Kiran 2016), steroid use (Ostenfeld et al. 2015), NSAID-use (Hakkarainen et al. 2015) as well as patient’s comorbidities. However, the surgeon’s experience plays a vital role in the surgical outcome (Marinello et al. 2016). In our study, during all colorectal surgeries, an experienced surgeon (> 30 resections with anastomosis per year) was a member of the operating team and, we believe, that this pre-requisite had a major impact on successful surgical outcomes in our series.

Approximately 35% to 44% of all patients with a stoma will never get a chance for stoma reversal as they do not wish to be re-operated or due to their poor health conditions (Maggard et al. 2004). This fact makes a multi-stage procedure less popular and, currently, colorectal surgeons prefer a multi-modal but single-stage colorectal resection with primary anastomosis (Awotar et al. 2017). As a part of a multi-modal single-stage procedure, the fecal-loaded colon is cleaned by intraoperative colonic or bowel lavage prior to primary anastomosis (Hong et al. 2017). In our multimodal fail-safe model, we performed on-table bowel irrigation through the efferent ileal loop after performing the anastomosis with saline in case of a rectal resection (Herzberg et al. 2022) or emergency procedures.

Independent of location and its surgical technique, the anastomotic site should not have circulatory compromise, tension or intraluminal stress. Perioperatively a macroscopic perfusion test is commonly performed, however, a frequent mistake is often made due to peripheral perfusion testing. In our approach, we performed a bleeding test immediately at the edge of the resection margins. Generally, during end-to-end stapling anastomosis, the proximal as well as the distal colon is often freed from fatty tissue. This could result in decreased perfusion of the anastomotic site (Shekarriz et al. 2015). We also avoided sharp-angled edges —so called dog-ears— of the remnant rectum that could lead to insufficient blood supply (Kuramoto et al. 2017). Recently, indocyanine green (ICG) fluorescence imaging is introduced for potential of reducing AL due to insufficient blood supply. Current clinical non-randomized controlled trials have shown a reduction of the AL rate in colorectal surgery by using ICG (Blanco-Colino and Espin-Basany 2018).

For the ileocolic side-to-side anastomosis, we performed continuous hand-sewn anastomoses with additional seams on the edges. These seams not only relieved tension on the anastomosis, but also provided a mechanical strain relief on mobile intestine. The use of hand-sewn anastomoses in right side resections might cause a higher rate of AL in this group, as there is an individual learning curve for each surgeon performing this anastomosis. For the colorectal or coloanal anastomoses, a full mobilization of the splenic flexure up to the middle colic artery was performed. Anastomoses were always performed using a circular stapler, which was passed transanally. Before performing an end-to-end anastomosis, the sphincteric muscle was stretched for at least 3 min to reduce the risk for anal fissures. These steps lead to an early passage of stools and return of bowel activity and prevent an increased intraluminal pressure due to constipation of stool (Shigeta et al. 2016). Several studies including the German Guidelines for Colorectal cancer (Deutsche Krebsgesellschaft et al. 2019) have recommended the creation of a colonic pouch for functional optimization and to lower AL rates in colorectal resections (Pucciarelli et al. 2019). However, we performed end-to-end anastomosis in colorectal resections, which led to fewer sutures or stapling lines needed for reconstruction and, additionally, allowed the use of over-the-scope-clip (OTSC) or endosponge® in the case of AL. This facilitated the reconstruction of the rectal wall. Following the straight anastomosis in our cohort, we noticed a lower AL rate in comparison to the reported data in all non-straight reconstructions (Pucciarelli et al. 2019). We can deduce that a comprehensive perioperative protocol and rigorous surgical steps resulted in better surgical outcomes in our study.

Positioning of the stapler spine for circular stapling next to the linear stapler line to prevent long overlapping of both stapler lines is already well described as the so-called double-stapling technique (Cohen et al. 1983).

Intraoperative sigmoidoscopy was performed routinely in our series for colorectal and coloanal anastomosis, with the advantages for identifying that the anastomosis is complete, performing an air test, and identifying any bleeding from the anastomosis. In case of a leakage, oversewing of the anastomosis at the site of leakage is a common approach (Lanthaler et al. 2008); however, it is associated with higher postoperative leakage rates compared with a new set up of the anastomosis (Ricciardi et al. 2009). Therefore, in case of any irregularity during intraoperative sigmoidoscopy, a new anastomosis must be set up. This occurred once during the study period.

### Postoperative settings

Since the introduction of the “fast-track” concept by Kehlet et al. in 1999, a multimodal rehabilitation program has accelerated patients’ postoperative recovery (Kehlet and Mogensen 1999) and is still part of the modern Enhanced Recovery after Surgery (ERAS) protocols (Gustafsson et al. 2019). In this perspective, early nutritional support is considered beneficial in reducing the incidence of serious postoperative infectious complications (Rana et al. 1992). According to the ERAS guidelines, the positive effect of an early nutritional support is crucial for recovery. Sharma et al. have shown promising results with an additional oral nutritional supplementation after colorectal surgery (Sharma et al. 2013). According to commonly applied ERAS concepts, we believe that the positive effect of an early nutritional uptake is crucial for recovery. However, it is customary to understand that an anastomosis needs to heal uncomplicated. Therefore, intraluminal stress should be avoided as much as possible. In our series, we used a strictly low-volume high-calorie diet for all patients during the postoperative period. Compared to the modern ERAS concepts, our study with this diet has shown promising results.

We routinely performed an early endoscopic anastomosis assessment on 4th day after colorectal or coloanal anastomosis. Although endoscopic assessment is risky for low anastomosis, various imaging studies have shown low sensitivity and high false negative rates for early detection of AL (Sparreboom et al. 2016). Historically, the treatment of choice for a leaking colorectal or coloanal anastomosis had been resection of the anastomosis with a Hartmann’s procedure (Chereau et al. 2018). Pelvic abscesses had often to be drained percutaneously using a CT- or ultrasound-guided approach (Meylemans et al. 2018). With the fail-safe approach, intraoperative positioning of a drainage tube near anastomosis, as well as on-table lavage over an efferent loop of the ileostomy prevented the development of a pelvic abscess. In the case of an AL after low colorectal anastomoses, various management strategies are available (Blumetti and Abcarian 2015). We prefer an application of the OTSC rather than endoscopic stenting or Endosponge® therapy, which essentially enlarges the defect during its initial placement. Indications for use of the OTSC system are small defects less than 1.5 cm in size and an absence of pelvic collection (Arezzo et al. 2012). Therefore, an early endoscopic anastomosis assessment and early detection of AL is an essential component of treatment as the defect is more likely to close (van Koperen et al. 2009). Using OTSC, rates of anastomotic healing from 86% (Arezzo et al. 2012) to over 90% have been reported (Haito-Chavez et al. 2014). Even the mean length of hospital stay was longer in the fail-safe model compared to the data reported by Arrick et al.(Arrick et al. 2019), the step towards this adoption might be justified by the reduced rate of AL in this fail-safe model.

### Limitations

A major limitation of this study is being a single-center non-randomized retrospective cohort study. The data of the control group was collected retrospectively, while the data of the study group was collected prospectively. Due to the design of a cohort study, the size of the fail-safe group is larger than the control group. Even if the population is comparable in terms of malignant diseases and demographics, the inclusion of right- and left-sided tumors and rectal cancers provides a group of diverse diseases with different surgical approaches. Comparing the results of the historical control group, it should be kept in mind that some of the positive trends observed in the study group were secured due to the technical innovations in laparoscopic surgery. The impact of each perioperative change adapted in the fail-safe model for the reduction of AL remains unclear. Further research is essential that can determine the efficacy of some controversies including mechanical bowel irrigation.

## Conclusion

Our study presents promising results of a multimodal fail-safe model for colorectal resections. The fail-safe approach comprehensively interlaces preoperative and postoperative settings with operative technical steps. Although not every single step is evidence-based, the application of a standard protocol resulted in a reduction of colorectal surgical complications. The overall rate of AL, including emergencies and extended multivisceral resections was 3.7% (*n* = 19) and 1.2% for elective rectal resections (*n* = 1/83). Our multimodal fail-safe model offers a structured perioperative protocol with standardized steps, even if some of these steps don’t corroborate with common surgical practice. Additionally, its application in emergencies not only reduces surgical complication rates but might even improve short- and long-term oncological outcomes. However, further investigations including new technologies like ICG imaging must be undertaken for fail-safe colorectal surgeries.

## Data Availability

The datasets generated and analyzed during the current study are not publicly available but are available from the corresponding author on reasonable request.
